# Molding Process Retaining Gold Nanoparticle Assembly Structures during Transfer to a Polycarbonate Surface

**DOI:** 10.3390/polym16111553

**Published:** 2024-05-31

**Authors:** Philipp Zimmermann, Daniel Schletz, Marisa Hoffmann, Patrick T. Probst, Andreas Fery, Jürgen Nagel

**Affiliations:** 1 Institut für Polymerwerkstoffe, Leibniz-Institut für Polymerforschung Dresden e.V., Hohe Straße 6, D-01069 Dresden, Germany; zimmermann-philipp@ipfdd.de; 2 Institut für Physikalische Chemie und Physik der Polymere, Leibniz-Institut für Polymerforschung Dresden e.V., Hohe Straße 6, D-01069 Dresden, Germany; schletz@ipfdd.de (D.S.); probst@gold.kobe-u.ac.jp (P.T.P.); 3 Department of Electrical and Electronic Engineering, Graduate School of Engineering, Kobe University, Kobe 657-8501, Japan

**Keywords:** molding, nanoparticle, immobilization, plasmonic properties, polycarbonate

## Abstract

The immobilization of gold nanoparticle (AuNP) linear surface assemblies on polycarbonate (PC) melt surface via molding is investigated. The order of the particle assemblies is preserved during the molding process. The assemblies on PC exhibit plasmonic coupling features and dichroic properties. The structure of the assemblies is quantified based on Scanning Electron Microscopy (SEM) and image analysis data using an orientational order parameter. The transfer process from mold to melt shows high structural fidelity. The order parameter of around 0.98 reflects the orientation of the lines and remains unaffected, independent of the injection direction of the melt relative to the particle lines. This is discussed in the frame of fountain flow during injection molding. The particles were permanently fixed and withstood the injection molding process, detachment of the substrate, and extraction in boiling ethanol. The plasmonic particles coupled strongly within the dense nanoparticle lines to produce anisotropic optical properties, as quantified by dichroic ratios of 0.28 and 0.52 using ultraviolet–visible–near-infrared (UV–Vis–NIR) spectroscopy. AuNP line assemblies on a polymer surface may be a basis for plasmonic devices like surface-enhanced Raman scattering (SERS) sensors or a precursor for nanowires. Their embedding via injection molding constitutes an important link between particle-self-assembly approaches for optically functional surfaces and polymer processing techniques.

## 1. Introduction

Some metallic nanoparticles exhibit plasmonic [1] properties, i.e., the light-induced collective oscillation of conduction electrons confined to nanometer size. The spectral position and shape of this plasmon resonance can be tailored and enhanced by material composition, particle size, and particle arrangement [2]. Various applications that take advantage of the resulting strong localized electric fields, hot carrier generation, and other properties, e.g., in solar cells [3] or sensors [4], require the robust immobilization of the particles on solid surfaces. While there is a profound understanding of various approaches towards forming such functional structures [5] on surfaces like glass, silica, or silicon wafers, as well as their integration into polydimethylsiloxane (PDMS) elastomer, [6] thermoplastic parts, which would be particularly suitable for the applications mentioned above, remain widely unexplored. Their manufacture is inexpensive and they often possess favorable properties such as low weight and design flexibility [7]. Therefore, there is an urgent need for bridging the gap between techniques for surface assembly of functional particle structures and polymer processing.

A key step for establishing advanced functionalities like controlled field enhancement, circular dichroism, or metamaterial effects is controlling particle order in non-close packed layers. A set of nano-patterning methods, which have developed considerably in recent years, are based on capillary force-driven self-assembly [8] and confinement assembly [9] of nanoparticles into designed, non-close-packed arrays. These methods are capable of producing large-area particle arrangements in the form of periodic lines [10], 2D arrays [11], or more complex structures with control down to the single-particle level [12,13]. As lithography-free, low-cost templates to produce periodic particle lines, wrinkled PDMS templates are frequently used [14]. Furthermore, these templates allow transfer printing of ordered structures onto substrate surfaces like glass or silicon wafers [15]. The key question addressed in this manuscript is how patterns formed by confinement assembly can be immobilized on a polymer surface using injection molding techniques.

In previous works, disordered silica [16], gold [17], and calcite [18] nanoparticles were successfully transferred to plastic surfaces during injection molding or by 3D printing. The particles were applied in advance to a substrate or mold surface and subsequently brought into contact with a thermoplastic melt. As a result, plastic surfaces with partly embedded nanoparticles were obtained. Parts of the transferred particle surfaces were still accessible for small molecules, as we demonstrated by the catalytic effect of embedded AuNPs [17].

Recently, we demonstrated that this transfer process is also suitable for ordered AuNP structures [19]. A plastic surface furnished with an AuNP assembly fabricated with our transfer process exhibited an enhancement factor of 10^4^ in SERS experiments. However, although the potential of this new material was revealed in a proof of concept experiment, details of the process are not known or understood.

The emphasis of this paper is the detailed investigation of the transfer process during injection molding. The orientation and optical properties of linear periodic single-line AuNP assemblies before and after partial embedding in PC are evaluated. For this purpose, array assemblies of AuNPs were fabricated on silicon wafers as substrates and transferred to PC parts via injection molding. The influence of the melt flow direction with respect to the AuNP line orientation on the reproduction quality is explored. In addition, the robustness of the immobilized nanoparticle structures is demonstrated by means of boiling ethanol extractions.

## 2. Materials and Methods

### 2.1. Materials

Ascorbic acid (AA, >99%), hydrogen tetrachloroaurate (HAuCl4, >99.9%), Linear poly (ethylenglycol)methyletherthiol (PEG-SH, molar weight 6 kDa), sodium borohydride (NaBH4, 99%), and Hexadecyltrimethylammoniumbromid (CTAB) were obtained from Sigma Aldrich SE (Steinheim, Germany). Hexadecyltrimethylammonium chloride (CTAC, >99%) was obtained from Molekula GmbH (München, Germany). PC Makrolon 2407 was purchased from Bayer AG(Leverkusen, Germany). Ethanol 99.8% was purchased from VWR International LLC (Darmstadt, Germany). All chemicals and solvents were used as received and purified water (Milli-Q-grade, 18.2 MΩ cm at 25 °C) was used in all preparations.

### 2.2. Synthesis of Spherical AuNP

The synthesis described here was adopted from [19]. First, Wulff seeds were synthesized by tempering an aqueous CTAB solution containing HAuCl_4_ for at least 10 min. Under vigorous stirring, NaBH_4_ was added and the seeds obtained were aged for 30 min. Afterward, CTAC and ascorbic acid were added to the previously prepared Wulff seed solution. Then, a mixture of CTAC and HAuCl_4_ was added. After 15 min, the solution was centrifuged, washed with water, and redispersed in CTAC solution. Next, the prepared solution was mixed with CTAC and ascorbic acid. Then, HAuCl_4_ was added continuously with a syringe pump. Again, the solution was centrifuged, washed, and redispersed in CTAC to obtain AuNPs in the solution.

### 2.3. Functionalization of the AuNP Surface with PEG Ligands

The functionalization described here was adopted from [19]. For the functionalization with PEG-SH ligands (Sigma Aldrich SE (Steinheim, Germany); 6 kDa), DCM with PEG-SH was added to an aqueous AuNP solution. After adding 1 mL methanol, the solution was shaken overnight in an automated shaker to secure complete ligand exchange. Finally, the PEG-grafted AuNPs were centrifuged, washed, and redispersed in water.

### 2.4. Assemblies on Substrates

Adopted from previous work [15], wrinkled templates were made in a multistep process. First, Sylgard 184 PDMS (Sigma Aldrich SE Steinheim, Germany) was mixed with a cross-linker and cured inside a polystyrene dish. Subsequently, the PDMS was cut into 1 cm × 4.5 cm strips. To generate a template, 1 of those strips was fixed in a homebuilt stretching apparatus, elongated by 40%, and O_2_-plasma-treated. After the procedure, the strip was slowly relaxed and cut into 1 cm × 1 cm pieces to obtain a wrinkled PDMS template.

For the template-assisted assembly, a solution of PEG-coated AuNPs was prepared by mixing MQ water, ethanol, and PEG-grafted AuNP solution. A cleaned silicon wafer was hydrophilized in oxygen plasma and used as a substrate for the assemblies. Next, the PEG-grafted AuNP solution (see above) was deposited on the substrate. Right away, the PDMS template was put on top, with the wrinkled surface facing the substrate. After drying, the PDMS template was removed. This led to AuNP chain assemblies on the substrate.

### 2.5. Transfer by Injection Molding

A substrate with AuNP assembly was fixed on a magnetic stainless steel plate with Capton adhesive tape and then mounted in the rectangular cavity (length 68 mm × width 34 mm × depth 2 mm) of the mold of a DEMAG ergotech 80–200 compact (Sumitomo (SHI) Demag Plastics Machinery GmbH, Nürnberg, Germany) injection molding machine. The orientation of the AuNP line assemblies on the substrate was either parallel or perpendicular with respect to the injected melt flow direction. The mold temperature was held at 120 °C. The mold was then closed and thermoplastic melt of type Makrolon 2407 (Bayer AG, Leverkusen, Germany) was injected with standard parameters (injection pressure about 400 bar, injection velocity about 70 mm/s, and hold pressure about 200 bar). The temperature of the PC melt was set to 305 °C. Thereby, the melt came in contact with the assemblies mounted on the mold wall. After solidification, the substrate, the stainless steel plate, and the PC part were taken together from the mold. The substrate was removed from the molded PC part, leaving the transferred AuNP assembly on the PC surface. For evaluating the robustness against particle bleeding, the PC parts were placed in a Soxhlet apparatus and extracted with boiling ethanol for 4 h. Subsequently, the samples were dried in a vacuum oven.

### 2.6. SEM and Orientation Analysis 

SEM images were acquired with a Phenom microscope (FEI Company, Hillsboro, OR, USA) operated at an accelerating voltage of 10 kV in low vacuum mode (pressure 60 Pa). The Backscatter detector (BSD) was used. The stitching mode was used to acquire wide-area SEM images of AuNP assemblies on the original substrates. SEM images of the AuNP assemblies on the original substrates, after transfer to PC and after extraction with ethanol, were taken to assess the line orientation of the AuNP assemblies. Matching positions (ROI) were measured at all 3 stages of the sample history (see Figure S3 in the Supporting Information). Using the image analysis program ImageJ (version 1.53p) and the plugin OrientationJ (version 2.0.4), the angle-dependent orientation of the AuNP lines was determined from each SEM image. From these data, the 2-dimensional nematic order parameter was calculated for all 3 stages of the sample. A detailed description of the orientation analysis and the calculation of the order parameter can be found in Figure S4 in the Supporting Information.

### 2.7. Optical Spectroscopy

Optical measurements were carried out with a Cary 6000i UV–VIS–NIR Spectrophotometer (Agilent Technology, Santa Clara, CA, USA) equipped with a Thompson Polarizer for linear polarization in transmission configuration. For the entire wavelength range, a halogen lamp was used as the light source. For each polarization angle, a baseline measurement was taken against air.

## 3. Results and Discussion

### 3.1. Sample Concept

**Figure 1:**
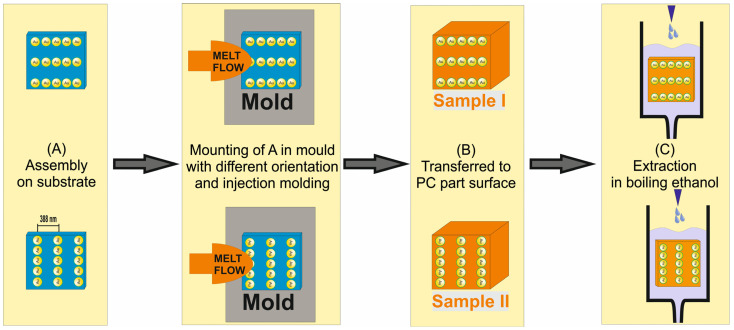
Scheme of sample preparation. Spherical AuNPs, surface functionalized with PEG in single-line confinement assembly on silicon substrate, were mounted in the tool of an injection molding machine. (**A**) Assemblies on substrates. The substrates were mounted in the mold with orientation of the AuNP lines parallel (for Sample I) or perpendicular (for Sample II) to the melt flow. (**B**) PC parts of Samples I and II, respectively, after removing the substrates. Parts were turned downside up in the scheme to view the particle assemblies. (**C**) Extraction in ethanol of both types of samples. The assemblies on the substrates, on the PC part after molding, and after extraction were characterized.

The main goal of this contribution is to investigate the effect of melt flow orientation on the immobilization and structural integrity of linearly aligned nanoparticle assemblies transferred to the surface of a thermoplastic material. Experiments were carried out according to the scheme in Figure 1 as follows: Spherical AuNPs functionalized with a PEG shell were deposited on silicon wafers as substrates, templated by a wrinkle-structured silicone rubber. Silicon wafers were chosen as substrates because they exhibit an atomically flat surface, fit in the tool, and withstand rapid heating and cooling during the subsequent injection molding process. The template wrinkle geometry was chosen so that the AuNPs were arranged on the substrate as single-particle lines with an average line spacing of 388 nm. The distance of particles within a line was defined by the size of the PEG molecules on the surface of the AuNPs and was approximately 1.5 nm. The synthesis of the AuNPs as well as the confinement assembly were described previously [19]. The particle diameter was about 80 nm. A modified injection molding process was used to transfer and immobilize the assemblies on a plastic surface. To this end, the substrates with the linear assemblies on their surfaces were mounted in the cavity of an injection molding tool, and then the PC melt was injected. The melt front moved from the injection point along the mold until the entire cavity was filled. From a macroscopic point of view, the direction of melt flow is parallel to the inserted substrate surface (see Figure 1). This poses a risk for the aligned nanoparticles to be displaced due to shearing. This effect becomes especially relevant when the melt flow is perpendicular to the nanoparticle lines.

Therefore, in this paper, the influence of the flow direction of the melt, parallel or perpendicular to the particle lines, on the structural integrity of the transferred nanostructures is investigated. The orientation of the AuNP lines on the formed PC parts as well as the stability of the particle embedding is discussed.

### 3.2. Orientation Analysis of AuNP Lines Aligned Parallel to the Melt Flow Direction (Sample I)

In this experiment, a silicon substrate with AuNP single-line assembly on its surface was inserted in the molding tool so that the lines were oriented parallel to the melt flow direction (Sample I in Figure 1). After the injection of a PC melt and its solidification, the PC part was released from the substrate. The AuNP line assembly was characterized on the original substrate after transfer to PC via injection molding and after extraction in ethanol. All of the SEM images show the identical region of interest (ROI).

**Figure 2:**
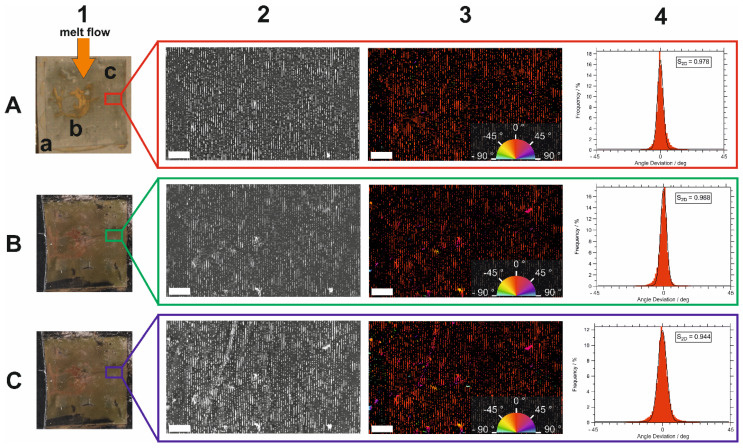
Orientation analysis of Sample I. Identical ROI of the same AuNP line assembly on (**A**) original substrate, (**B**) PC after injection molding, and (**C**) PC after ethanol extraction, with AuNP lines aligned parallel to the melt flow direction; (1) photographs of AuNP assembly on the original substrate, with direction of melt flow (orange arrow) and Sites A, B, and B (**A1**), on PC (**B1**) and on PC after ethanol extraction (**C1**); (2) SEM images of identical ROI of the same AuNP lines on surfaces of 1; (3) color-coded SEM images of 2, where color represents orientation angle of the nanoparticle lines; (4) distributions of orientation angle deviation (α − α_ref_) from reference direction, extracted from SEM images in 3, with calculated 2D order parameters S_2D_. Photos and SEM images of (**B**,**C**), respectively, were mirrored with respect to (**A**) to get the same orientation. Scale bars are 5 µm.

With the naked eye, one can distinguish three different sites on the original substrate surface, see Figure 2-A1. Site A exhibited no optical differences compared to the plane substrate, thus, no AuNPs were deposited here. Site B exhibited a golden shine due to the non-selective deposition of gold multilayers. The grey areas in Site C, however, comprise AuNP lines with close contact of AuNPs within a line and with large gaps between lines. Such regions of aligned particle arrays are prevalent in all samples. All three types of areas were found on the original substrate (A) and on the PC part before (B) and after (C) extraction in ethanol. (see Figure S1 for SEM images of Sites A, B, and C of AuNP line assembly on PC after extraction in ethanol). This already suggested a good reproduction of assemblies, independently of the type of assembly. The characterization is focused on sites of Type C in the following discussion because they show well-controlled plasmonic properties.

To evaluate the order and orientation of the AuNP lines, SEM images of the assemblies on the original substrate surface were taken, as shown in Figure 2-A2. The SEM image shows AuNP lines with few defects running from the top to the bottom of the image. To characterize the alignment of the lines, an orientation analysis in accordance with [20] was performed using ImageJ with the plugin OrientationJ. In this process, a local orientation angle *α* was calculated for each pixel of the SEM image based on the gradient of gray values in its proximity. This local orientation angle was color-coded, see Figure 2-A3.

We evaluated the orientation angles of all pixels in the SEM image to obtain a direct quantitative measure of orientation. The orientation frequency is shown in Figure 2-A4. The reference angle was *α_Ref_* = 0°, i.e., parallel to the AuNP line direction. The AuNP lines with an orientation angle of *α* = 0° were color-coded in red, i.e., they were oriented strongly parallel to each other. Single particles and line defects, on the other hand, showed a range of orientation angles and were coded in multiple colors. A Gaussian distribution of the orientation angles described the distribution well with a maximum at *α_Max_* = *α_Ref_* = 0*°*. From the averaged deviation *α* − *α_Ref_*, the nematic 2D order parameter S_2D_ was calculated by Equation (1) [21].

(1) $$S_{2D}=\langle 2\;cos^{2}\;(\alpha_{i}-\alpha_{Ref})-1\rangle$$

S_2D_ indicates the orientation angle deviation from the reference angle and can take values between 1 for maximum orientation at *α_Max_ = α_Ref_* and 0 for isotropic orientation.

To calculate the orientation angle distribution, the Gaussian distribution was applied with a local window of 8 pixels, a minimum energy of 5%, and a Coherence threshold of 20%. This allows the orientation of single particles and regions where the orientation could not be analyzed reliably to be excluded from the distribution. As shown in Figure 2-A4, the order parameter *S*_2*D*_ for the line assemblies on the original substrate was 0.978, indicating a high level of alignment of the particle lines along *α_Ref_*, with only a small number of defects due to the preparation.

An equivalent analysis of SEM data was performed with AuNP assemblies transferred to the PC part surface (see Figure 2-B). Exactly the same ROI was identified for this analysis. For these assemblies, the order parameter *S*_2*D*_ = 0.988 (see Figure 2-B4), indicating a high order, equivalent to that at the same ROI on the original substrate. The small difference was a result of the SEM image qualities, particularly of sharpness and resolution. The important conclusion is, that the orientation and order of the AuNP lines have not changed due to the injection molding process, i.e., by melt flow and detaching the original substrate from the PC part.

The PC part was extracted in boiling ethanol and finally analyzed using the same procedure in ImageJ, see Figure 2-C1–4. Here, the order parameter *S*_2*D*_ calculated from the distribution in Figure 2-C4 was 0.944, pointing to a high orientation of the lines along *α_Ref_*. The value is only 0.034 lower than that on the original substrate. Consequently, neither the injection molding process nor the hot ethanol extraction changed the order of the AuNP lines significantly when the melt flow was parallel to the AuNP lines.

**Figure 3:**
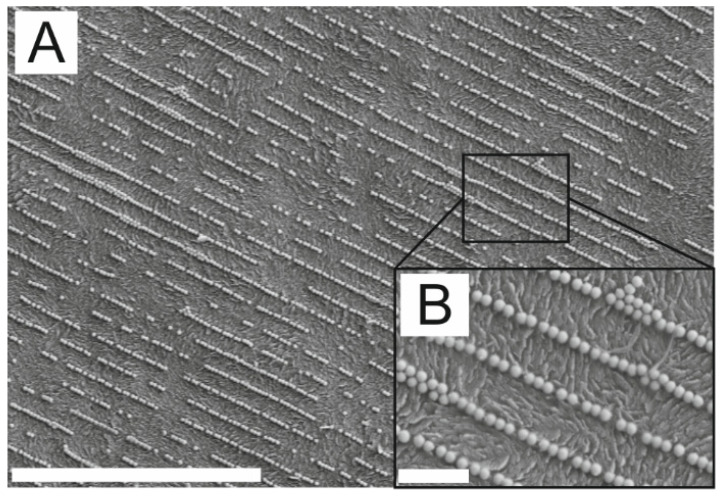
(**A**) SEM images of AuNP line assembly on PC after ethanol extraction, with AuNP lines aligned parallel to the melt flow direction. (**B**) Section from A with higher magnification. Scale bars are 5 µm for (**A**) and 0.5 µm for (**B**).

In order to get more detailed insights into the state of the AuNP assembly on PC after the ethanol extraction, high-resolution SEM images were recorded. The image in Figure 3-A shows a typical assembly of AuNPs arranged in single lines. The entire surface of the PC is not covered by AuNPs, but there are also free spots between the lines. Also, a few imperfections such as AuNPs arranged in double lines and non-linear line segments can be found. Overall, the SEM image in Figure 3-A is a typical example of the arrangement of AuNP single lines, which can be found in the same manner on wafers after the confinement assembly. In Figure 3-B, a section of the image in A with higher magnification is shown. The individual AuNPs are easily recognizable and exhibit a spherical shape with a diameter of around 70 nm. This corresponds to the appearance of the particles on the wafer before the transfer to the PC. Consequently, neither the injection molding process nor the ethanolic extractions caused any changes to the particle geometry. In addition to the arranged AuNPs, a rough PC surface is clearly visible. Injection-molded PC samples normally have a smooth surface. Therefore, the surface roughness must have been caused by the ethanol extraction. Although the extraction in ethanol might cause swelling and de-swelling of the surface layer of the PC itself, the assemblies were not affected. This speaks in favor of the AuNPs being permanently fixed on the surface of the PC part.

**Figure 4:**
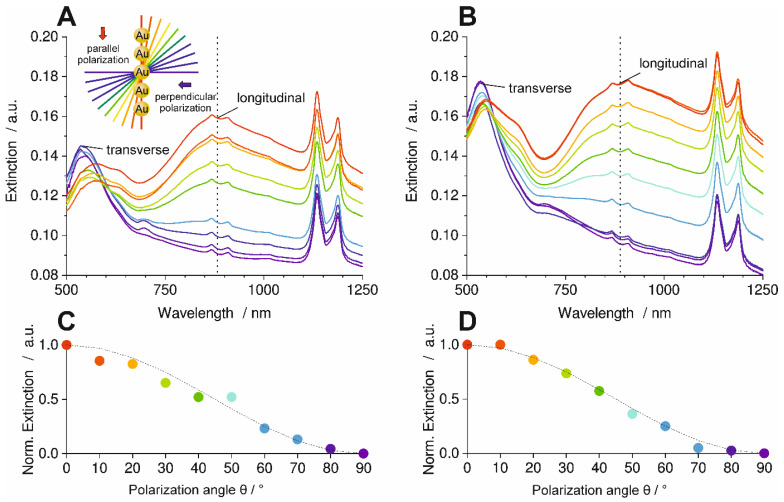
Polarization-dependent optical spectra of AuNP line assemblies on PC, Sample I. (**A**) after molding and (**B**) after hot ethanol extraction. AuNP lines aligned parallel to the melt flow direction. Strong anisotropies can be seen at the extinction of the longitudinal mode (dotted lines in (**A**,**B**)) of the optical spectra as a function of the polarization angle in relation to the orientation of the AuNP lines. The anisotropy of the extinction intensity of the longitudinal mode follows a cos^2^ dependence ((**C**,**D**), dotted line). Y-axis in (**A**,**B**) starts at 0.08. The colors of the points in C and D correspond to the color of the spectra in A and B.

The orientation analysis by SEM has already shown that the AuNP line assembly retains its original orientation after transfer to the PC part via injection molding and after extraction in ethanol. Optical spectroscopy with polarized light was applied to investigate the plasmonic properties of the particles within the lines, which serves as a benchmark for assembly quality over larger areas. Sample I was mounted between the polarizer and the detector of an optical spectrometer. Spectra were recorded at 10 different polarization angles varying from 0° (red graph) to 90° (purple graph) relative to the orientation of particle lines in transmission mode against air as a reference (Figure 4). A specially designed sample holder was used to ensure the same sample positioning for both measurements (Sample I after molding and after ethanol extraction).

The polarized optical spectra in Figure 4-A show bands at 868, 909, 1134, and 1186 nm, which were assigned to the PC (see Figure S2 for optical spectra of an injection molded PC part without AuNPs in the Supporting Information). For polarization parallel to the line assemblies (red graph), the longitudinal plasmon mode was excited most efficiently as a broad band at 908 nm. The additional mode around 633 nm can be assigned to a subradiant longitudinal mode where the particle dipoles are not all parallel to each other. [22] Polarization perpendicular to the line assemblies (purple graph) produces a transversal resonance at 546 nm, whereas the longitudinal modes disappear. This optical anisotropy with selective excitation of transversal or longitudinal plasmon modes highlights the high quality of AuNP line assemblies over areas of 100 mm^2^ that were preserved in the molded PC part. Consequently, the molding process did not affect the plasmonic interactions in the assembly.

To quantify the optical anisotropy, the dichroic ratio *DR* is calculated for the longitudinal band from the extinction values *E* with Equation (2).

*DR* = (*E*_0°_ − *E*_90°_)/(*E*_0°_ + *E*_90°_) (2)

This amounts to *DR* = 0.28 for the line assembly transferred to PC in Figure 4-A. The polarization-dependent optical spectra of Sample I after extraction in ethanol are shown in Figure 4-B. The relation between the different spectra is essentially the same as in Figure 4-A. For this sample, *DR* was calculated as *DR* = 0.3. This is close to the value of Sample I after molding. The graphs in Figure 4-C,D show the maximum normalized extinction values of the longitudinal band for each measured polarization angle. The data points of both graphs followed a cos^2^θ function (dotted lines), which is typical for dipolar plasmonic modes [22]. Slight deviations in the data points of both graphs could be attributed to the used polarizer, which was operated by hand. This shows that the particle lines are not degraded through the injection molding process or the subsequent boiling ethanol extraction and that they retain their strong plasmonic coupling within the lines. This was ensured by the partial embedding of the AuNPs in the PC part surface for permanent immobilization.

### 3.3. Orientation Analysis of AuNP Lines Perpendicular to the Flow Direction (Sample II)

**Figure 5:**
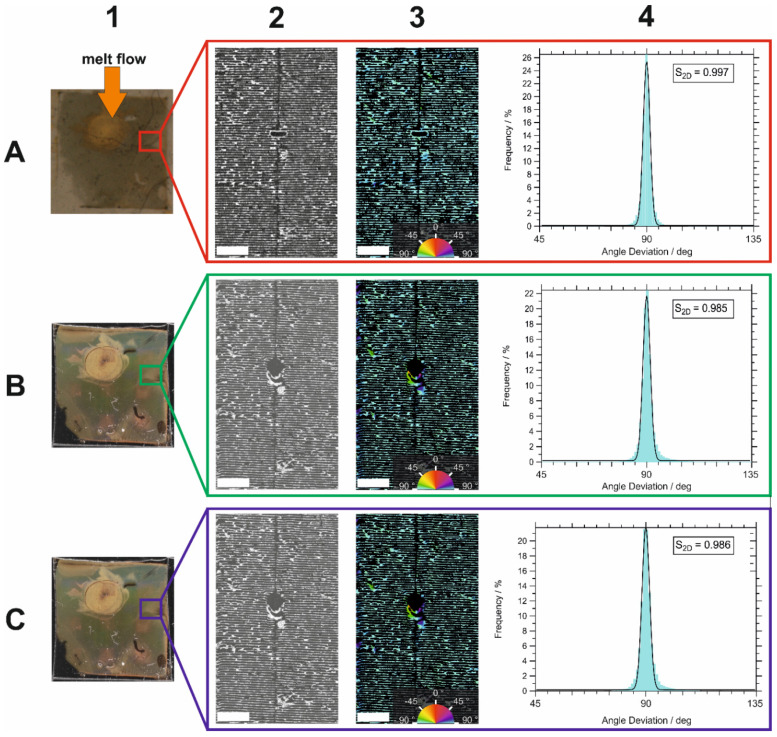
Orientation analysis of Sample II. Identical ROI of the same AuNP line assembly on (**A**) original substrate, (**B**) PC after injection molding, and (**C**) PC after ethanol extraction, with particle lines aligned perpendicular to the melt flow direction; (1) photographs of AuNP assembly on the original substrate, with direction of melt flow (orange arrow) (**A1**), on PC (**B1**), and on PC after ethanol extraction (**C1**); (2) SEM images of identical ROI of the same AuNP lines on surfaces of 1; (3) color-coded SEM images of 2, where color represents orientation angle of the AuNP lines; (4) distributions of orientation angle deviation (α − α_ref_) from reference direction extracted from SEM images in 3 with calculated 2D order parameter S_2D_. Photos and SEM images of (**B**,**C**), respectively, were mirrored with respect to (**A**) to get the same orientation. Scale bars are 5 µm.

In this experiment, the substrate was inserted in the cavity of the injection molding machine with the orientation of the AuNP lines perpendicular to the melt flow direction (Sample II in Figure 1). Figure 5 shows photos of the assembly on the original substrate and on the PC after molding and after hot ethanol extraction as well as SEM images and orientation distribution of the exact same ROI.

Like Sample I, the photos of the assembly on the original substrate, on PC, and after extraction in ethanol in Figure 5-A1,B1,C1, respectively, show numerous similarities in their appearance. The SEM image of the original substrate in Figure 5-A2 shows many horizontally aligned particle lines with mostly minor defects. A larger defect in the form of an ellipse appeared in the center of the image. The color-coded SEM image of orientation analysis in Figure 5-A3 shows that most of the particle lines are oriented at an angle of 90°. Again, the nematic orientation parameter S_2D_ was calculated using the formula described in Equation (1). However, here *α_Ref_* = 90° was used, as this was the dominant orientation of the particle lines. This resulted in a value of *S*_2*D*_ = 0.997 for the orientation of the AuNP lines on the substrate, which corresponds to a very strong orientation along α*_Ref_*.

In the SEM image in Figure 5-B2, an enlargement of the centrally located defect can be observed. Presumably, some AuNP line segments have been displaced by the melt during injection molding. This is clearly visible in the color-coded image in Figure 5-B3. However, as this displacement occurred mainly at the few defects present, the S_2D_ value of 0.985 is only slightly below that of the AuNP lines on the original substrate. Like Sample I, the orientation of the assembly was only slightly affected by the injection molding process, although the orientation of the lines in Sample II was perpendicular to the melt flow. After ethanol extraction, no differences are observed in either Figure 5-C2,C3 or Figure 5-B2,B3, respectively. The calculated S_2D_ was 0.986, which nearly equals the S_2D_ of the PC part after molding. Here, no visible changes in the assembly occurred as a result of the extraction. More detailed SEM images of Figure 5-C2 showed the same typical appearance of the AuNP assembly shown in Figure 3.

**Figure 6:**
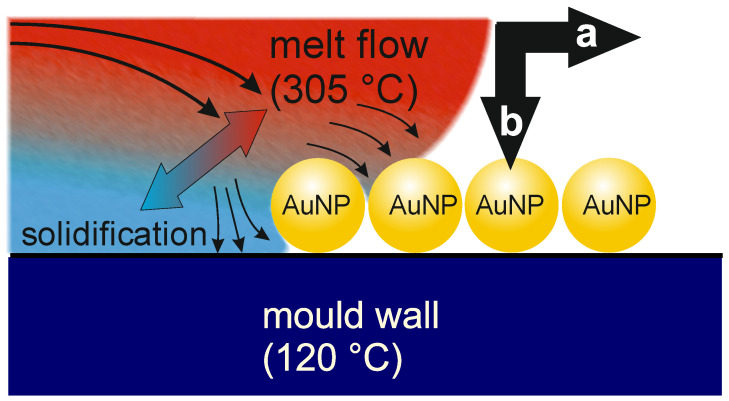
Scheme of the melt flow conditions during injection molding. The arrows indicate the flow direction of the fountain flow of the melt in the center of the cavity and the flow vector of a melt volume segment approaching a particle near the mold wall. a = vector of melt flow in the flow center, b = vector of the melt flow close to the mold surface.

The statement that no significant changes in the structure of the AuNP line assemblies occurred, independently of the orientation of the lines relative to the flow direction, is remarkable at first glance. One would expect a shift of particles along the flow direction of the melt. This would lead to a change in interparticle distance and reorientation of lines in the case of parallel melt flow, or in bending of the lines in the case of perpendicular melt flow. However, we did not observe this. It only occurred rarely at structural defects. To understand this behavior, the characteristic of flowing melt is considered. A thermoplastic melt is a high-viscous liquid. As such, it exhibits a lamellar flow in the mold cavity. The melt is at a much higher temperature than the mold wall. In our case, the mold temperature is below the glass transition temperature of the PC. Thus, as soon as the PC melt contacts the mold wall, a small melt volume segment immediately cools down and freezes. This leads to a velocity gradient between the center of the cavity and the wall, where the velocity equals zero. In contrast, a melt segment on the melt front in the center of the cavity has a high temperature and high velocity. Successional injected melt moves this segment towards the wall surface, whereby the flow vector is normal to the wall surface. This is the typical situation during fountain flow, as shown in Figure 6. Thus, for small particles, velocity components in the main direction of the melt flow are negligible, and the small particles remain in place. Probably, larger particles may be affected by this component. However, it was not observed for the nanoparticles used here. In the case of two small particles in close vicinity, the melt could spread on the particle surface if the interfacial energy between particle and melt is high, thereby pushing the particles apart. However, a breaking down of the assembly structure was not observed. Probably, rapid cooling of the melt upon contact with the mold wall may have resulted in freezing of the melt, which conserved the assembly structure. Only for stacked particles in the observed defect structures that impose higher friction on the melt, the influence of the melt flow is not negligible, which can facilitate particle displacement. Overall, the assembly structures on the substrate are almost completely reproduced during transfer to the PC part by injection molding, independent of the flow direction of the melt.

**Figure 7:**
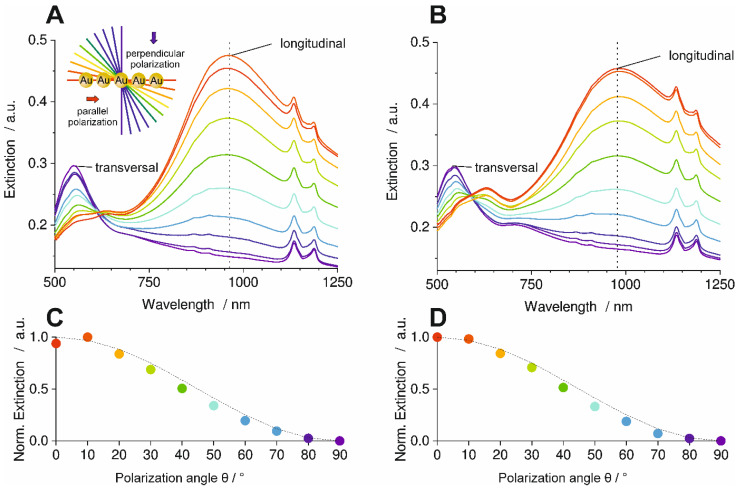
Polarization-dependent optical spectra of AuNP line assemblies on PC, Sample II. (**A**) After molding and (**B**) after hot ethanol extraction. AuNP lines aligned perpendicular to the melt flow direction. Strong anisotropies can be seen at the extinction of the longitudinal mode (dotted lines in (**A**,**B**)) of the optical spectra as a function of the polarization angle in relation to the orientation of the AuNP lines. The anisotropy of the extinction intensity of the longitudinal mode follows a cos^2^ dependence ((**C**,**D**), dotted line). Y-axis in (**A**,**B**) starts at 0.13. The colors of the points in C and D correspond to the color of the spectra in A and B.

Optical spectra of Sample II were recorded with polarized light of different polarization angles after molding as well as after boiling ethanol extraction in the same way as for Sample I. The resulting spectra are shown in Figure 7.

The spectra in Figure 7-A show multiple bands that are assigned to the PC, like in Figure 4. The transversal plasmon resonance band of the AuNP appears at 0° polarization (perpendicular to the AuNP lines) and is located at 549 nm. The band intensity decreased with the increasing polarization angle, whereas the longitudinal band aroused with a maximum at 960 nm. The longitudinal mode is the most intense at a polarization angle of 90°, i.e., polarization parallel to the AuNP lines. The anisotropic behavior of the particle lines is clearly demonstrated by the relation of the intensities of the transversal and longitudinal bands.

Increasingly parallel polarization of the light again reveals a subradiant longitudinal mode at 639 nm, as already described for Sample I. The dichroic ratio *DR*, calculated by Equation (2) for the longitudinal band, was *DR* = 0.52.

The spectra of the PC part after boiling ethanol extraction in Figure 7-B are almost equal to that of the PC part after molding, pointing to a permanent and stable immobilization of the AuNP assemblies. The dichroic ratio calculated for the longitudinal band was *DR* = 0.47, only slightly smaller than that of the part after molding. Thus, the ethanol extraction did not change order and orientation significantly. This finding is similar to that of Sample I.

The maximum extinction of the longitudinal band as a function of the polarization angle follows a cos^2^θ function again for both the part after molding and the ethanol-extracted part (Figure 7-C,D).

## 4. Conclusions

In this work, AuNP line assemblies were fabricated on silicon wafers as substrates, guided by topographical templates. These assemblies were successfully transferred via injection molding onto the surfaces of PC parts. During injection molding, the assemblies were contacted with a PC melt, where the direction of the melt flow was parallel or perpendicular to the orientation of the AuNP lines, respectively. Calculated *S*_2*D*_ order parameters of the AuNP line assemblies before and after injection molding were as high as *S*_2*D*_ = 0.978 and 0.988, respectively, for parallel injected melt and *S_2D_* = 0.997 and 0.986, respectively for perpendicular injected melt. Interestingly, the high orientation of the AuNP line assemblies was almost completely reproduced during the molding transfer independently of the orientation of the AuNP lines with respect to the melt flow. Polarization-dependent optical spectra of the transferred assemblies revealed optical anisotropy with selective excitation of transversal or longitudinal plasmon modes, even after extracting the samples in boiling ethanol. Calculated dichroic ratios of the longitudinal mode before and after ethanol extraction revealed nearly identical values with *DR* = 0.28 and 0.3, respectively, for parallel injected melt and *DR* = 0.52 and 0.47, respectively, for perpendicular injected melt. Not only was the microscopic order maintained, but also the plasmonic properties of the AuNP assemblies. This pointed to a conservation of the assembly structure even on the sub-nanometer scale. The reason for that is the successful partial embedding of the AuNP lines in the melt and the PC part, respectively. The fixation withstands even extraction in boiling ethanol. The structures may be used as SERS sensors, in catalysis, and plasmonic metamaterials. The method of robust immobilization of ordered nanoparticle structures on polymer surfaces during injection molding can be easily extended to other particle types, assembly structures, and thermoplastics.

## Data Availability

Data are contained within the article and Supplementary Materials.

## Supplementary Materials

The following supporting information can be downloaded at https://www.mdpi.com/article/10.3390/polym16111553/s1, Figure S1. SEM images of site a, b and c of AuNP line assembly on PC after extraction in ethanol. Figure S2. Polarization-dependent optical spectra of an injection molded PC part (without AuNP). Figure S3. Generating identical ROI out of SEM images of the same AuNP line assembly on different surfaces. Figure S4. SEM image processing for orientation analysis and evaluation. References [23,24,25,26,27,28] are cited in Supplementary Materials.
