# Supplementary material for: Molding Process Retaining Gold Nanoparticle Assembly Structures during Transfer to a Polycarbonate Surface
Source: Polymers (Basel). 2024 May 31;16(11):1553. doi: 10.3390/polym16111553 (PMC11174599; doi:10.3390/polym16111553)
Supplement: Supplementary file 1 [file polymers-16-01553-s001.zip › polymers-3004058-supplementary.pdf]

Supporting Information

Molding Process Retaining Gold Nanoparticle Assembly Structures during Transfer to a Polycarbonate Surface

Philipp Zimmermann [1], Daniel Schletz [2], Marisa Hoffmann [2], Patrick T. Probst [2,3], Andreas Fery [2,*] and Jürgen Nagel [1,*]

[1] Institut für Polymerwerkstoffe, Leibniz-Institut für Polymerforschung Dresden e.V., Hohe Straße 6, D-01069 Dresden, Germany
[2] Institut für Physikalische Chemie und Physik der Polymere, Leibniz-Institut für Polymerforschung Dresden e.V., Hohe Straße 6, D-01069 Dresden, Germany
[3] Department of Electrical and Electronic Engineering, Graduate School of Engineering, Kobe University, Kobe 657-8501, Japan
* Correspondence: fery@ipfdd.de (A.F.); nagel@ipfdd.de (J.N.)

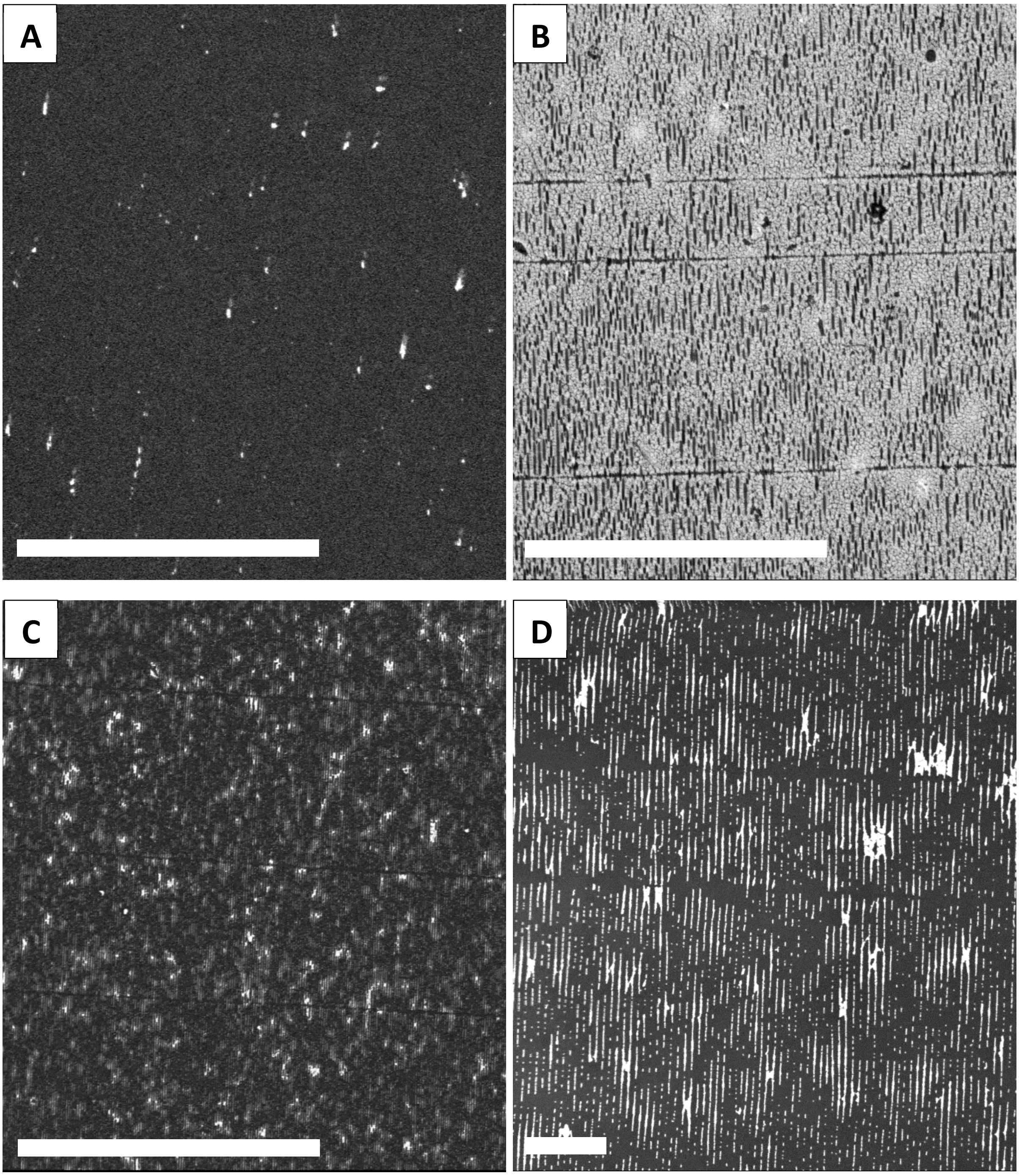

Figure S1. SEM images of site a, b and c of AuNP line assembly on PC after extraction in ethanol. A) SEM image of site a. No AuNP line assembly is visible. B) SEM image of site b. Multilayers of agglomerated AuNP are visible. C) SEM image of site c. The whole area is covered with AuNP lines. D) SEM image of site c with higher magnification. Scale bars are 50 µm for A, B and C and 5 µm for D.

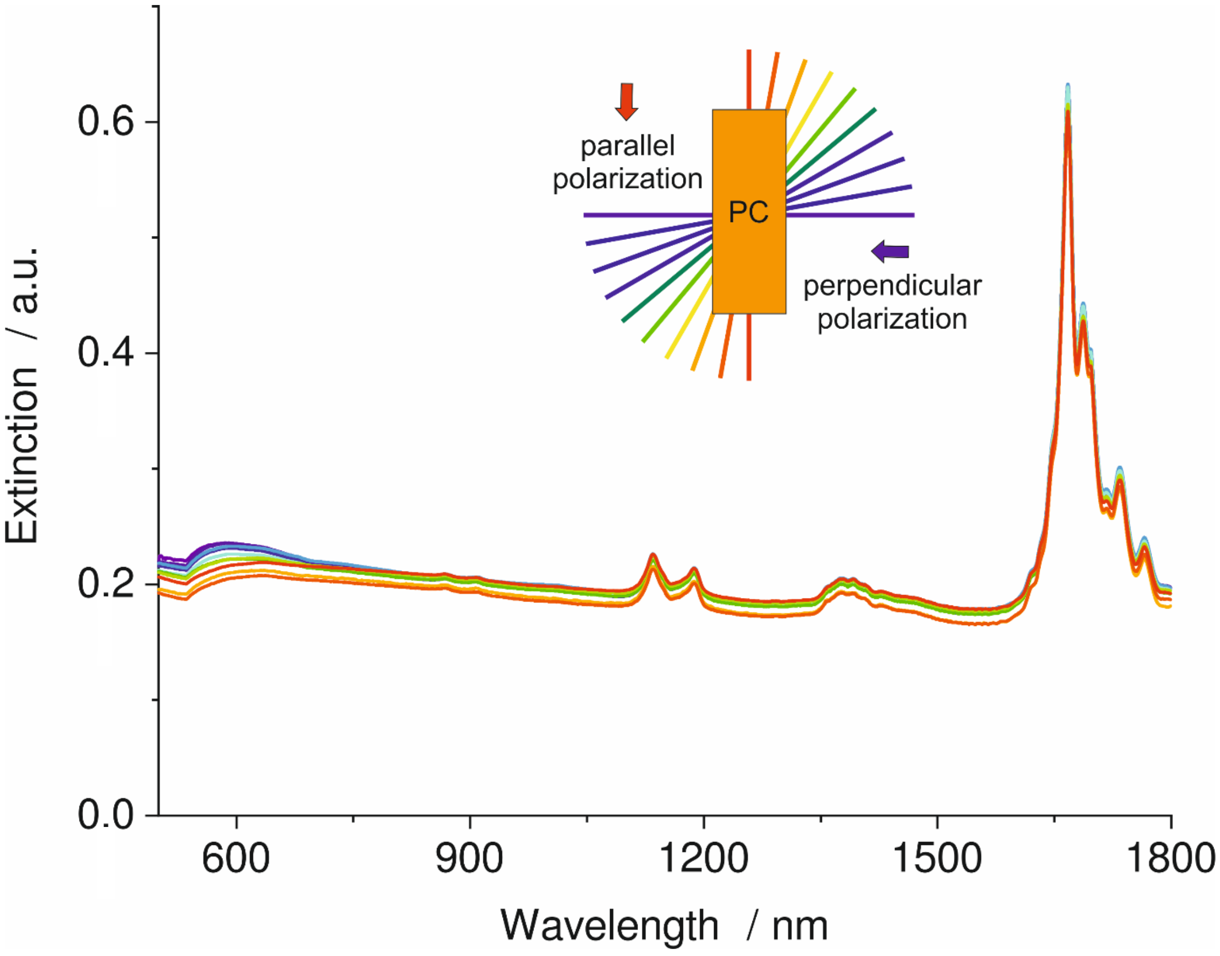

Figure S2. Polarization-dependent optical spectra of an injection molded PC part (without AuNP). Spectra were recorded at 10 different polarization angles varying from 0° (red) to 90° (blue) in transmission mode against air as reference. All spectra show bands at 868, 909, 1134 , 1186, 1384, 1667, 1687, 1697, 1717, 1734 and 1765 nm which were assigned to PC.

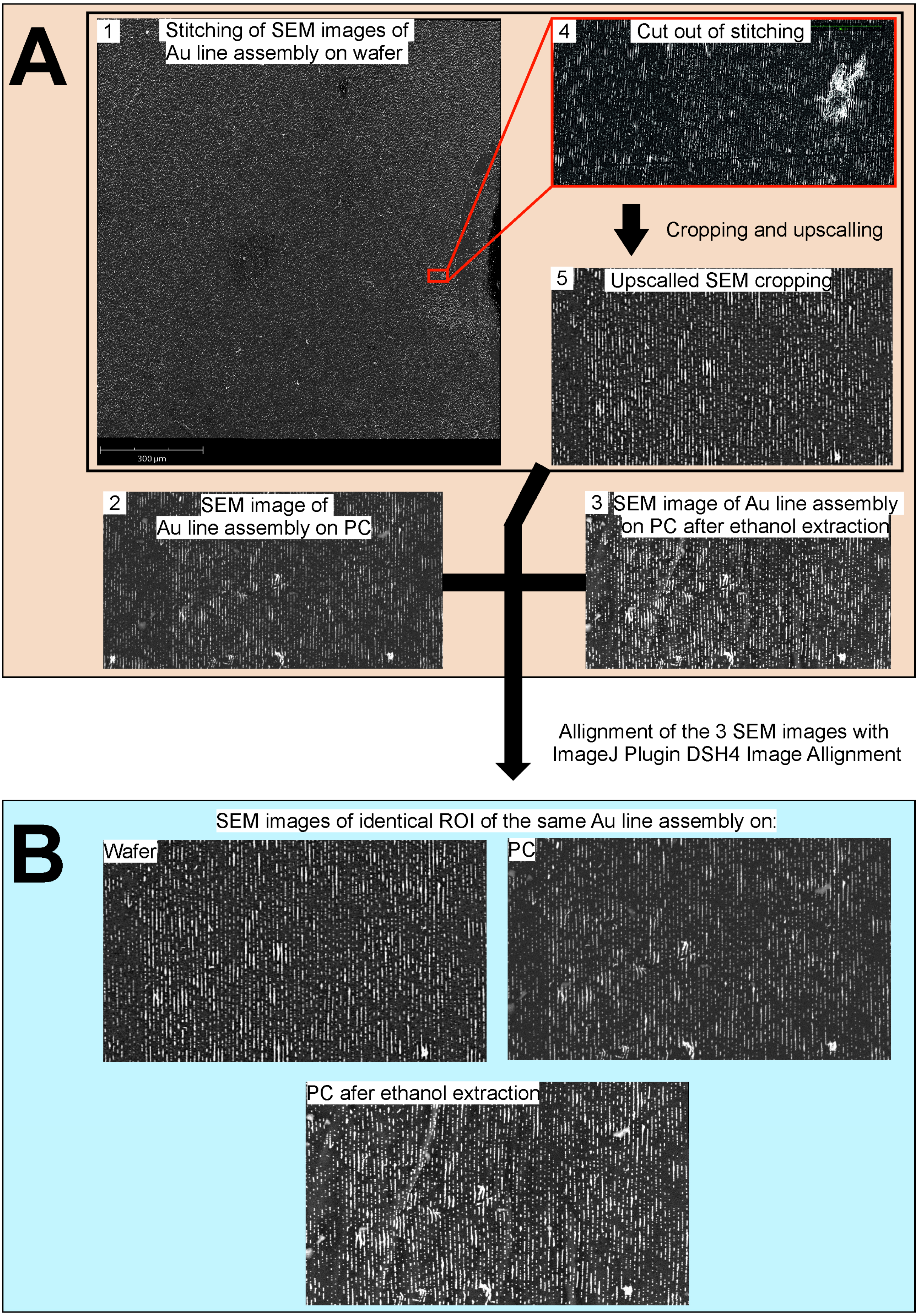

Figure S3. Generating identical ROI out of SEM images of the same AuNP line assembly on different surfaces. A) For the AuNP line assembly on wafer, a large area of several square millimeters was examined by SEM. The individual SEM images were stitched together to form the stitching A1. For the AuNP line assembly on PC and on PC after ethanol extraction, SEM images A2 and A3 were acquired at high magnification at the same measurement position and mirrored horizontally. This

measurement position was then cropped from the SEM stitching (A4) and scaled up (A5) with an enhanced deep residual network [23]. **B)** Using the ImageJ plugin DSH4 [24], the three SEM images from A were aligned to obtain an identical region of interest (ROI) for orientation analysis.

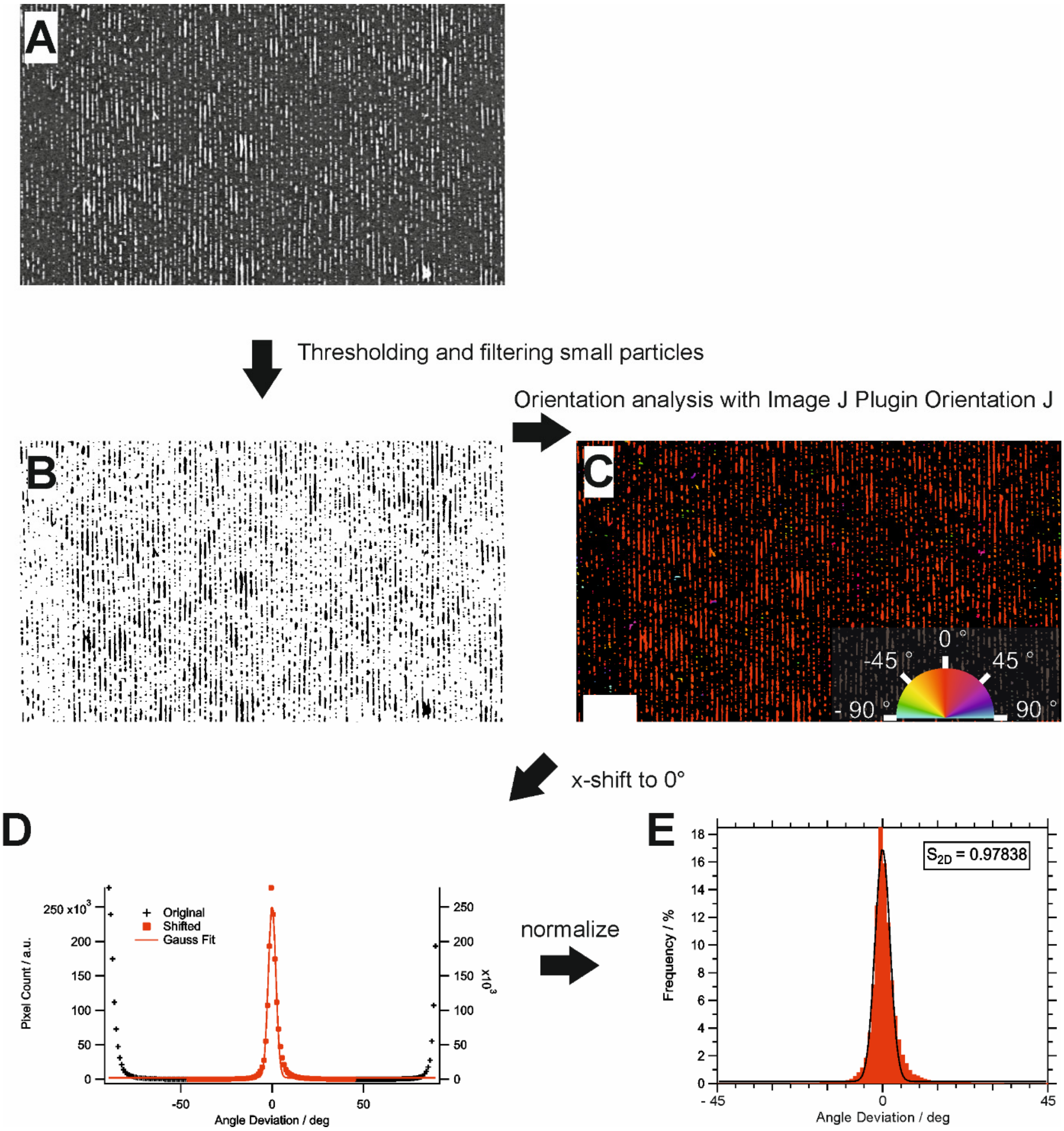

Figure S4. SEM image processing for orientation analysis and evaluation A) SEM image prepared according to the methodology of Figure S2. **B)** Binary SEM image of A. Conversion of the SEM image into a binary image with ImageJ [25, 26] by applying a threshold with default settings. Image noise was removed by applying a median filter with a pixel radius of 0. AuNP lines are displayed in black, the background in white. **C)** Performing orientation analysis of AuNP lines using the ImageJ plugin OrientationJ [27, 28]. A Gaussian gradient with a local pixel window of 8 px. was used for analysis. The local orientation of each pixel is shown as a false color image. The predominant orientation of AuNP lines on wafer was set as reference orientation angle (0° for parallel melt flow and 90° for perpendicular melt flow). The orientation angle can be read from the reference scale embedded in the image. **D)** Histogram of local orientation angle distribution from C. Only results with a minimum energy of 20% and a minimum coherence of 5% were considered. Since the orientation of the AuNP lines should correspond to a theoretical reference angle of 0°, the maximum of the distribution curve was shifted in X direction to 0°. **E)** Normalized distribution curve of D with the calculated average nematic 2D order parameter S_{2D}. The distribution curve in D was normalized and then the average nematic 2D order parameter S_{2D} was calculated. For this purpose, the orientation distribution was transformed into values of $S_{2D,\,i} = \langle 2\cos^2(\alpha_i) - 1\rangle$ and the average S_{2D} was calculated. S_{2D} indicates the orientation angle deviation from the reference angle and can take values between 1 for maximally oriented at $\alpha_{Max} = \alpha_{Ref}$ and 0 for isotropic orientation.

References

23. Lim, B., et al. *Enhanced deep residual networks for single image super-resolution*. in *Proceedings of the IEEE conference on computer vision and pattern recognition workshops*.
24. Bulgarelli, J., et al., *Dendritic Cell Vaccination in Metastatic Melanoma Turns "Non-T Cell Inflamed" Into "T-Cell Inflamed" Tumors.* Frontiers in Immunology, 2019. **10**.
25. Abramoff, M., P. Magalhães, and S.J. Ram, *Image Processing with ImageJ.* Biophotonics International, 2003. **11**: p. 36-42.
26. Schneider, C.A., W.S. Rasband, and K.W. Eliceiri, *NIH Image to ImageJ: 25 years of image analysis.* Nature Methods, 2012. **9**(7): p. 671-675.
27. Püspöki, Z., et al., *Transforms and Operators for Directional Bioimage Analysis: A Survey*, in *Focus on Bio-Image Informatics*, W.H. De Vos, S. Munck, and J.-P. Timmermans, Editors. 2016, Springer International Publishing: Cham. p. 69-93.
28. Rezakhaniha, R., et al., *Experimental investigation of collagen waviness and orientation in the arterial adventitia using confocal laser scanning microscopy.* Biomechanics and Modeling in Mechanobiology, 2012. **11**(3): p. 461-473.
